# Weakly nonlinear focused ultrasound in viscoelastic media containing multiple bubbles

**DOI:** 10.1016/j.ultsonch.2023.106455

**Published:** 2023-05-27

**Authors:** Shunsuke Kagami, Tetsuya Kanagawa

**Affiliations:** Department of Engineering Mechanics and Energy, Degree Program of Systems and Information Engineering, University of Tsukuba, 1-1-1 Tennodai, Tsukuba 305-8573, Japan

**Keywords:** Focused ultrasound, Viscoelastic media, Bubbly liquids, Weakly nonlinear wave, KZK equation, HIFU

## Abstract

To facilitate practical medical applications such as cancer treatment utilizing focused ultrasound and bubbles, a mathematical model that can describe the soft viscoelasticity of human body, the nonlinear propagation of focused ultrasound, and the nonlinear oscillations of multiple bubbles is theoretically derived and numerically solved. The Zener viscoelastic model and Keller–Miksis bubble equation, which have been used for analyses of single or few bubbles in viscoelastic liquid, are used to model the liquid containing multiple bubbles. From the theoretical analysis based on the perturbation expansion with the multiple-scales method, the Khokhlov–Zabolotskaya–Kuznetsov (KZK) equation, which has been used as a mathematical model of weakly nonlinear propagation in single phase liquid, is extended to viscoelastic liquid containing multiple bubbles. The results show that liquid elasticity decreases the magnitudes of the nonlinearity, dissipation, and dispersion of ultrasound and increases the phase velocity of the ultrasound and linear natural frequency of the bubble oscillation. From the numerical calculation of resultant KZK equation, the spatial distribution of the liquid pressure fluctuation for the focused ultrasound is obtained for cases in which the liquid is water or liver tissue. In addition, frequency analysis is carried out using the fast Fourier transform, and the generation of higher harmonic components is compared for water and liver tissue. The elasticity supresses the generation of higher harmonic components and promotes the remnant of the fundamental frequency components. This indicates that the elasticity of liquid suppresses shock wave formation in practical applications.

## 1 Introduction

The combination of ultrasound and bubbles has been utilized in a wide range of medical applications, for both treatment and diagnosis [1]. In tumor ablation therapy, the thermal effects radiated by oscillating bubbles improve the heating effect of high-intensity focused ultrasound (HIFU) [2], [3], [4], [5], [6], [7], [8], [9], [10]. Shock wave lithotripsy and histotripsy therapies use the oscillation or collapse of cavitation bubbles induced by the mechanical effects of HIFU to eradicate kidney stones and cancer cells [11], [12], [13], [14], [15], [16], [17], [18], [19], [20]. Ultrasound also induces the oscillation or collapse of bubbles injected into blood vessels during drug delivery to brain cancer cells, which opens the blood–brain barrier [21]. Moreover, bubbles encapsulated by lipid shells improve the resolution of images [22], [30], [31], [23], [24], [27], [25], [26], [28], [29] for ultrasound imaging as a real-time diagnostic technique.

For these medical applications, the investigation of bubble dynamics in soft viscoelastic media like human body is essential. Newtonian fluid is the most famous model in the field of fluid dynamics, but only viscosity is considered. The Kelvin–Voigt [32], [33], [34], [35], [36], [38], [19], [37], [39], [30], [31] and Zener models [41], [42], [45], [46], [43], [44], [47] have been used as mathematical models of liquid with viscoelasticity. The Kelvin–Voigt model corresponds to the generalized Newtonian fluid with the elasticity introduced while the relaxation effect is omitted. Yang and Church [34] investigated a behavior of single spherical bubble based on the Kelvin–Voigt model and Keller–Miksis equation [34], [52], [41], [35], [42], [36], [38], [19], [43], [37], [44], [39], [40], and showed that liquid elasticity decreases the nonlinearity of bubble oscillation. Keller–Miksis equation is a model of bubble dynamics in compressible and viscoelastic liquid and often used in combination with Kelvin–Voigt model [34], [35], [36], [38], [39], [40]. Murakami et al. [37] developed a model of the behavior of a non-spherical single bubble based on the Kelvin–Voigt model and Rayleigh–Plesset equation and investigated the shape stability of bubbles. Rayleigh–Plesset equation is the most famous model of bubble dynamics, but is the limiting case of Keller–Miksis equation assuming the incompressible liquid. Qin et al. [39] investigated the effects of the liquid viscoelasticity and the interaction of two bubbles on behavior of each bubble using the Kelvin–Voigt model and Keller–Miksis equation. The Maxwell viscoelastic model [48], [49], [50], [51] is also the generalized Newtonian fluid with the relaxation effect of viecoelastic medium, however the elastic term is omitted. Fogler and Goddard [48] investigated the collapse of bububle in viscoelastic media based on the Maxwell model and Rayleigh–Plesset equation.

Further, the Zener model corresponds to the generalized Kelvin–Voigt and Maxwell model, including the elastic term and the relaxation effect of a viscoelastic medium. Warnez and Johnsen [42] developed a numerical method for bubble behavior based on the Zener model and Keller–Miksis equation, and they found that incorporating the relaxation time increases bubble growth. Zilonova et al. [46] investigated the interaction of two bubbles with the effects of drag force and translation based on the Zener model and Gilmore–Akulichev equation [53]. Gilmore–Akulichev equation has broader applications than the Keller–Miksis equation [46], [47]. Filonets and Solovchuk [47] investigated the single bubble behavior excited by a dual-frequency ultrasound based on the Zener model and Gilmore–Akulichev equation, and they showed that the elasticity of a liquid increases the threshold pressure of bubble collapse.

Therefore, the behavior of a single or few bubbles in viscoelastic liquid has been well investigated; however, considering multiple bubbles from a macroscopic view is necessary for practical medical applications. The Westervelt equation [54] for full-wave propagation and the Khokhlov–Zabolotskaya–Kuznetsov (KZK) equation [55], [56] for quasi-planar propagation have both been used as continuum models for the nonlinear propagation of focused ultrasound. The KZK equation corresponds to the limiting form of the Westervelt equation and has been widely used [57], [58], [59], [60], [61], [62], [63], [64], [65], [66], [67], [68], [69], [70], [71], [72], [73], [74], [75], [77], [78], [76], [79], [80], [81], [82], [83], [84], [85], [86], [87] as a computational model for medical applications, owing to its accuracy and usability in numerical calculations. However, the original KZK equation [55], [56] was derived for propagation in single phase liquids without bubbles.

The authors were involved in the first derivation of the KZK equation for a liquid containing multiple bubbles [88], [89], [90], using theoretical analysis based on volumetric averaged basic equations for liquids containing multiple bubbles. Kagami and Kanagawa [10] introduced the thermal effects of gas inside the bubbles into the KZK equation and obtained numerical solutions. However, these models [88], [89], [90], [10] only considered viscosity and neglected the elasticity of the liquid. Recently, Hasegawa et al. [91] succeeded in introducing the elasticity of a liquid into the weakly nonlinear wave equation; however, this model was limited to the spatial one-dimensional form and could not be used for focused ultrasound. In addition, the viscoelasticity of the bubble–liquid interface was considered, but not the viscoelasticity of the entire liquid. In summary, a mathematical model that can describe the liquid viscoelasticity of both the bubble–liquid interface and the entire liquid, the nonlinear propagation of focused ultrasound, and the oscillations of multiple bubbles would be very useful.

**Fig. 1:**
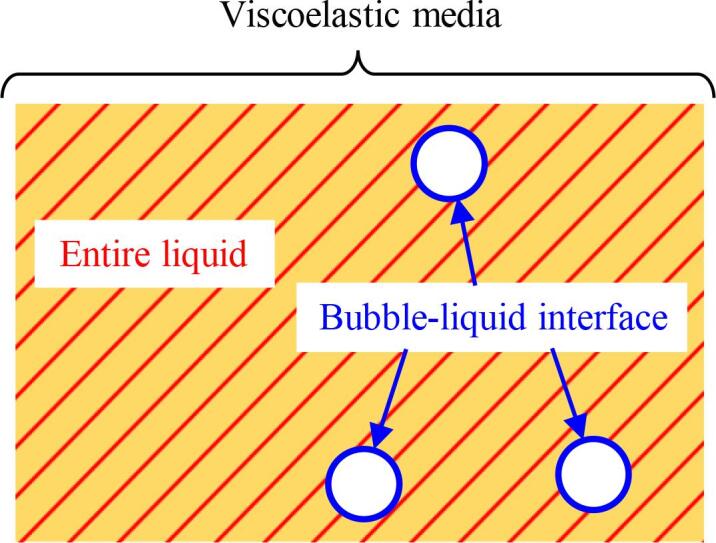
Regions in which viscoelasticity is considered.

In this study, the KZK equation describing the weakly nonlinear propagation of focused ultrasound in a viscoelastic liquid containing multiple bubbles is derived. In Section 2, volumetric averaged equations for a liquid containing multiple bubbles are introduced, based on the mixture model [96], [97], [98], [99]. The viscoelasticities of the entire liquid and bubble–liquid interface are introduced, as shown in Fig. 1. The momentum conservation equation in the mixture model is combined with the Zener model to incorporate the viscoelasticity of the entire liquid, and the Keller–Miksis equation for bubble dynamics is used to incorporate the viscoelasticity of the bubble–liquid interface. Theoretical analysis based on the perturbation expansion and multiple-scales method [121] is carried out in Section 3, and the derivation of the KZK equation is demonstrated. This KZK equation is composed of terms representing the nonlinear, dissipation, dispersion, and diffraction effects of ultrasound propagation. The effects of the liquid elasticities on the nonlinearity, dissipation, dispersion, and phase velocity of focused ultrasound and the natural frequency of bubble oscillation are investigated by comparing cases in which the liquid is water (without elasticities) or liver tissue (with elasticities). By a comparison among the Zener model, the Maxwell model, and the Kelvin–Voigt model, the effects of the rigidity and the relaxation time are compared. In addition, a comparison of the liquid viscoelasticity of the entire liquid and bubble–liquid interface is conducted. In Section 4, the numerical solution of the newly obtained KZK equation is presented. The spatial distribution of the fluctuation of liquid pressure for the focused ultrasound is obtained. The dispersion effect on the numarical solution appeared. In addition, frequency analysis is conducted using the fast Fourier transform (FFT), and the generation of higher harmonic components is compared for water and liver tissue. As a result, the elasticity of liver tissue supresses the generation of higher harmonic components and promotes the remnant of the fundamental frequency components compare to the case of water, although the maximum value of the rise of liquid pressure are quite close. This result implies that the elasticity of liquid suppresses shock wave formation in practical applications.

## 2 Formulation of the problem

### 2.1 Problem statement

The weakly nonlinear propagation of focused ultrasound in a liquid containing multiple bubbles is considered. In this study, the viscoelasticity of the liquid phase is considered in two regions: the entire liquid and the bubble–liquid interface (see Fig. 1).

**Fig. 2:**
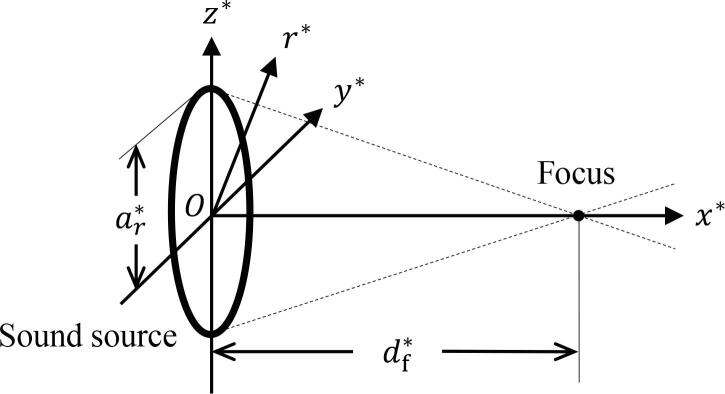
Schematic of spatial coordinates. Ultrasound radiated from a circular sound source with focused beam.

Focused ultrasound is radiated from a sound source in a viscoelastic medium containing multiple bubbles (Fig. 2). The center of the sound source is set as the origin. The $x^{*}$-axis represents the distance from sound source, and the $r^{*}$-, $y^{*}$-, and $z^{*}$-axes denote the distances from the $x^{*}$-axis. The following relation is provided:

(1) $$r^{*}=\sqrt{y^{*2}+z^{*2}}\text{.}$$

Note that the surface shape of the sound source is not only focusing, but can also be in a planar or spreading form, and the shape profile of the sound source is not only circular, but can also be rectangular or elliptical.

The KZK equation is derived by assuming quasi-plane propagation, i.e., weakly diffracted (focused or spreading) waves. In this study, two types of KZK equations are derived: two-dimensional (2D) and three-dimensional (3D) spatial forms, as in our previous work [10]. Although the 2D KZK equation is assumed for axial symmetric ultrasound, the 3D KZK equation can handle asymmetric propagation, such as ultrasound radiated by an elliptical or rectangular sound source or propagated in a nonuniform medium, such as the human body. The following relation is provided as a mathematical formula of the quasi-plane waves [88], [90], [93], [10].

(2) $$R_{0}^{*}\ll L^{*}\ll a_{i}^{*},$$

where $R_{0}^{*}$ is the initial bubble radius, $L^{*}$ is the typical ultrasound wavelength, and $a_{i}^{*}$ (i=r,y,z) are typical lengths in each direction. $a_{r}^{*}$ is set as the radius of circular sound source, and $a_{y}^{*}$ and $a_{z}^{*}$ are set as the long and short diameters of elliptic sound sources, or the width and height of rectangular sound sources, etc.

In the theoretical analysis, the following assumptions are introduced for simplicity.

(a-1)The elasticity of the liquid phase is considered; however, the viscoelastic shells [30], [31], [23], [27], [25], [26], [28], [29] coating the bubbles are not.
(a-2)The viscosity and elasticity of the gas phase are neglected because they are significantly smaller than those of the liquid phase.
(a-3)The basic equations based on the mixture model [96], [97], [98], [99] of bubbly liquids are used; [88], [94], [95], [93] those based on the two-fluid model [99], [100] were used in our previous work [88], [89], [90], [10] because the two-fluid model needs many assumptions and experimental laws to incorporate the viscoelasticity of the liquid phase. Note that the mixture model requires some assumptions such as (a-4) and (a-6) described below.
(a-4)The difference of velocity between the gas and liquid phases is sufficiently small, and then the drag force [102], [103] is not considered.
(a-5)The initial void fraction is sufficiently small.
(a-6)The viscosity and elasticity of the gas–liquid mixture are modeled by only those of the liquid phase, because the viscosity and elasticity of the gas phase are sufficiently smaller than those of the liquid phase according to Assumptions (a-2) and (a-5).
(a-7)The bubble–bubble interaction [104], [105], [106], [46], [44], [39], which may be dominant under high void fraction, is not considered.
(a-8)Bubble oscillation is spherically symmetric.
(a-9)Bubbles do not collapse, appear, or coalesce.
(a-10)The phase change [107], [108], [109] and the heat transfer [110] at the bubble–liquid interface is not considered.
(a-11)The distributions of the pressure and temperature of the gas inside the bubbles [101] are not considered; they are treated as the averages inside each bubble.
(a-12)The temperature of the liquid phase is assumed constant, whereas the temperature of the gas phase is assumed to fluctuate.
(a-13)At initial state, the liquid is at rest and spatially uniform, except for the spatial distribution of bubbles. The nonuniformity of the bubble size [111], [112], [113], [114], [115] is not considered. The spatial nonuniformity of the bubble distribution at the initial state is introduced [88], [90], [93] because bubbles are only used in focus in medical applications.

### 2.2 Basic equations

The conservation laws of mass and momentum based on the mixture model [96], [97], [98], [99] are used:

(3) $$\frac{\partial \rho^{*}}{\partial t^{*}}+\nabla^{*}\cdot (\rho^{*}u^{*})=0,$$

(4) $$\frac{\partial \rho^{*}u^{*}}{\partial t^{*}}+\nabla^{*}\cdot (\rho^{*}u^{*}u^{*})+\nabla^{*}p_{L}^{*}-\lambda_{v,L}^{*}\nabla^{*}\left(\nabla^{*}\cdot u^{*}\right)-\lambda_{e,L}^{*}\nabla^{*}\left(\nabla^{*}\cdot d^{*}\right)-\nabla^{*}\cdot \tau^{*}=0,$$

where $t^{*}$ is time, $\rho^{*}$ is density, $u^{*}$ is the velocity vector, $d^{*}$ is the displacement vector, $p^{*}$ is pressure, $\lambda_{v,L}^{*}$ is the second viscosity constant, $\lambda_{e,L}^{*}$ is the Lamé constant, and $\tau^{*}$ is the deviatoric stress tensor. The subscripts G and L mean the values of the gas and liquid phases, respectively. In Eq. (4), the pressure of the mixture is assumed to be that of the liquid phase [89], [94], [93]. The density of the gas phase is generally sufficiently small; hence, the density of the mixture is defined by the liquid density as follows:

(5) $$\rho^{*}\equiv (1-\alpha )\rho_{L}^{*},$$

where α is the void fraction

(6) $$\alpha =\frac{4}{3}\pi R^{*3}n^{*},$$

where $R^{*}$ is the bubble radius, $n^{*}$ is the bubble number density, and the following conservation law is imposed:

(7) $$\frac{\partial n^{*}}{\partial t^{*}}+\nabla^{*}\cdot (n^{*}u^{*})=0\text{.}$$

The conservation of mass in each bubble is

(8) $$\frac{\rho_{G}^{*}}{\rho_{G0}^{*}}={\left(\frac{R_{0}^{*}}{R^{*}}\right)}^{3}\text{.}$$

The second viscosity $\lambda_{v,L}^{*}$ and Lamé’s constant $\lambda_{e,L}^{*}$ can be rewritten as follows:

(9) $$\lambda_{v,L}^{*}=K_{v,L}^{*}-\frac{2}{3}\mu_{L}^{*},$$

(10) $$\lambda_{e,L}^{*}=K_{e,L}^{*}-\frac{2}{3}G_{L}^{*},$$

where $K_{v,L}^{*}$ and $K_{e,L}^{*}$ are the bulk viscosity and elasticity, respectively; $\mu_{L}^{*}$ is the viscosity; and $G_{L}^{*}$ is the rigidity (shear modulus). The bulk viscosity $K_{v,L}^{*}$ was set to zero based on the Stokes assumption [94], [93]. The physical quantities in Eqs. (9), (10) were originally that of the gas–liquid mixture; however, they have been substituted by that of the liquid phase from Assumption (a-6) in Section 2.1.

Eqs. (6), (8) are substituted into Eq. (7), Eq. (5) into Eqs. (3), (4)), and Eq. (9), Eq. (10), and $K_{v,L}^{*}=0$into Eq. (4) to obtain the following:

(11) $$\frac{\partial \alpha \rho_{G}^{*}}{\partial t^{*}}+\nabla^{*}\cdot (\alpha \rho_{G}^{*}u^{*})=0,$$

(12) $$\frac{\partial }{\partial t^{*}}[(1-\alpha )\rho_{L}^{*}]+\nabla^{*}\cdot [(1-\alpha )\rho_{L}^{*}u^{*}]=0,$$

(13) $$\frac{\partial }{\partial t^{*}}[(1-\alpha )\rho_{L}^{*}u^{*}]+\nabla^{*}\cdot [(1-\alpha )\rho_{L}^{*}u^{*}u^{*})]+\nabla^{*}p_{L}^{*}+\frac{2}{3}\mu_{L}^{*}\nabla^{*}\left(\nabla^{*}\cdot u^{*}\right)-\left(K_{e,L}^{*}-\frac{2}{3}G_{L}^{*}\right)\nabla^{*}\left(\nabla^{*}\cdot d^{*}\right)-\nabla^{*}\cdot \tau^{*}=0\text{.}$$

The velocity $u^{*}$ and displacement $d^{*}$ can be related as follows:

(14) $$u^{*}=\frac{Dd^{*}}{Dt^{*}},$$

where the Lagrange derivative $D/Dt^{*}$ is the differential operator

(15) $$\frac{D}{Dt^{*}}=\frac{\partial }{\partial t^{*}}+u^{*}\cdot \nabla^{*}\text{.}$$

The deviatoric stress tensor $\tau^{*}$ is based on the Zener viscoelastic model: [41], [42], [45], [46], [43], [44], [47]

(16) $$\tau^{*}+\lambda_{\mathrm{relax},L}^{*}\frac{D\tau^{*}}{Dt^{*}}=2\mu_{L}^{*}\frac{D\gamma^{*}}{Dt^{*}}+2G_{L}^{*}\gamma^{*},$$

where $\lambda_{\mathrm{relax},L}^{*}$ is the relaxation time of the liquid phase, $D\gamma^{*}/Dt^{*}$is the velocity gradient tensor, and $\gamma^{*}$ is the deformation gradient tensor. When $\lambda_{\mathrm{relax},L}^{*}=0$, Eq. (16) reduces to the Kelvin–Voigt viscoelastic model [32], [33], [34], [35], [36], [38], [19], [37], [39], [30], [31]. Further, when $\lambda_{\mathrm{relax},L}^{*}=0$ and $G_{L}^{*}=0$, Eq. (16) reduces to a model of Newtonian fluid and the theoretical results reduce to those of our previous studies [88], [90], [93], [10], [115], [114], [113], [102], [95], [94]. From Eqs. (13), (16), the effect of the viscoelasticity of the entire liquid is introduced.

The Keller–Miksis equation [34], [52], [41], [35], [42], [36], [38], [19], [43], [37], [44], [39], [40] describing spherical bubble oscillation in a compressible viscoelastic liquid is used:

(17) $$\left(1-\frac{1}{c_{L0}^{*}}\frac{DR^{*}}{Dt^{*}}\right)R^{*}\frac{D^{2}R^{*}}{Dt^{*2}}+\frac{3}{2}\left(1-\frac{1}{3c_{L0}^{*}}\frac{DR^{*}}{Dt^{*}}\right){\left(\frac{DR^{*}}{Dt^{*}}\right)}^{2}=\frac{1}{\rho_{L0}^{*}}\left(1+\frac{1}{c_{L0}^{*}}\frac{DR^{*}}{Dt^{*}}\right)\left(p_{G}^{*}-\frac{2\sigma^{*}}{R^{*}}+3q_{L}^{*}-p_{L}^{*}\right)+\frac{R^{*}}{\rho_{L0}^{*}c_{L0}^{*}}\frac{D}{Dt^{*}}\left(p_{G}^{*}-\frac{2\sigma^{*}}{R^{*}}+3q_{L}^{*}\right),$$

where $c_{L0}$ is the speed of sound in pure water and σ is the surface tension. The subscript 0 represents initial values. Note that the Keller–Miksis equation can be used for cases in which the Mach number is less than one [46], [47], and the Gilmore–Akulichev model [46], [47] can be used with larger Mach numbers. The variable $q_{L}^{*}$ is given by

(18) $$q_{L}^{*}=\int_{R^{*}}^{\infty }\frac{\tau_{r^{*\prime }r^{*\prime }L}^{*}}{r^{*\prime }}\;dr^{*\prime },$$

where $r^{*\prime }$ is the distance from the center of each bubble. Note that $r^{*\prime }$ is based on the spherical coordinate of each bubble and differs from $r^{*}$, which is based on the macroscopic coordinate as shown in Fig. 2. The balance of the normal stresses across the bubble–liquid interface is

(19) $$p_{G}^{*}-(p_{L}^{*}+P^{*})=\frac{2\sigma^{*}}{R^{*}}-3q_{L}^{*}\text{.}$$

To obtain the constitutive equation for $q_{L}^{*}$, the Zener Eq. (16) is integrated from the bubble–liquid interface to infinity: [41], [42], [47], [44], [38], [39]

(20) $$\lambda_{\mathrm{relax},L}^{*}\frac{Dq_{L}^{*}}{Dt^{*}}+q_{L}^{*}+\lambda_{\mathrm{relax},L}^{*}\frac{1}{R^{*}}\frac{DR^{*}}{Dt^{*}}{\left(\tau_{r^{\prime }r^{\prime }L}^{*}\right|}_{r^{\prime }=R^{*}}=\frac{1}{3}\left\{-\frac{4}{3}G_{L}^{*}\left[1-{\left(\frac{R_{0}^{*}}{R^{*}}\right)}^{3}\right]-4\mu_{L}^{*}\frac{1}{R^{*}}\frac{DR^{*}}{Dt^{*}}\right\}\text{.}$$

From Eqs. (17), (18), (19), (20), the effect of liquid viscoelasticity at the bubble–liquid interface is introduced.

The energy equation describing the thermal conduction at the bubble–liquid interface [116] is used: [94], [93], [95], [10]

(21) $$\frac{Dp_{G}^{*}}{Dt^{*}}=\frac{3}{R^{*}}\left[(\kappa -1)\lambda_{G}^{*}{\left(\frac{\partial T_{G}^{*}}{\partial r^{*\prime }}\right|}_{r^{*\prime }=R^{*}}-\kappa p_{G}^{*}\frac{DR^{*}}{Dt^{*}}\right],$$

where κ is the ratio of the specific heat of the gas phase, $\lambda^{*}$ is the thermal conductivity of the gas phase, and $T^{*}$ is the temperature. The temperature gradient $\partial T_{G}^{*}/\partial r^{*\prime }{|}_{R^{*}}$ is substituted into the following model [117], which is used for analysis.

(22) $${\left(\frac{\partial T_{G}^{*}}{\partial r^{*\prime }}\right|}_{r^{*\prime }=R^{*}}=\frac{5}{4}\frac{T_{0}^{*}-T_{G}^{*}}{R^{*}}$$

Note that many temperature gradient models have been proposed, such as Refs. [117], [118], [119], [120]; however, the model in Eq. (22) [117] derived the most appropriate numerical solution of the models in our previous work [10] and is used again in this study. To close the set of equations, the Tait equation of state for the liquid phase and the equation of state for an ideal gas are used:

(23) $$p_{L}^{*}=p_{L0}^{*}+\frac{\rho_{L0}^{*}c_{L0}^{*2}}{n}\left[{\left(\frac{\rho_{L}^{*}}{\rho_{L0}^{*}}\right)}^{n}-1\right],$$

(24) $$\frac{p_{G}^{*}}{p_{G0}^{*}}=\frac{\rho_{G}^{*}}{\rho_{G0}^{*}}\frac{T_{G}^{*}}{T_{0}^{*}},$$

where *n* is the material constant (e.g., n=7.15 for water).

### 2.3 Parameter scaling

The following scale parameters are introduced to consider the low frequency and long wave as in our previous studies [92], [88], [93], [94], [89], [10], using the dimensionless amplitude ∊(≪1):

(25) $$\frac{\omega^{*}}{\omega_{B}^{*}}\equiv O(\sqrt{∊})\equiv \Omega \sqrt{∊},$$

(26) $$\frac{R_{0}^{*}}{L^{*}}\equiv O(\sqrt{∊})\equiv \Delta \sqrt{∊},$$

(27) $$\frac{U^{*}}{c_{L0}^{*}}\equiv O(\sqrt{∊})\equiv V\sqrt{∊},$$

where $\omega^{*},L^{*}$, and $U^{*}$ are the typical angular frequency, wavelength, and propagation speed, respectively, and Ω,Δ, and *V* are the dimensionless constants of O(1).

The assumption of weakly diffracted waves given by Eq. (2) is rewritten as follows (i=r,y,z): [10]

(28) $$\frac{L^{*}}{a_{i}^{*}}\equiv O(\sqrt{∊})\equiv \Gamma_{i}\sqrt{∊},$$

where the dimensionless constant $\Gamma_{i}$ of O(1) represents the degree of diffraction for each direction.

The liquid viscosity $\mu_{L}^{*}$, rigidity $G_{L}^{*}$, bulk elasticity $K_{v,L}^{*}$, and relaxation time $\lambda_{\mathrm{relax},L}^{*}$ are nondimensionalized as

(29) $$\frac{\mu_{L}^{*}}{\rho_{L0}^{*}U^{*}L^{*}}\equiv O(∊)\equiv \mu_{L}∊,$$

(30) $$\frac{G_{L}^{*}}{\rho_{L0}^{*}U^{*2}}\equiv O(1)\equiv G_{L},$$

(31) $$\frac{K_{v,L}^{*}}{\rho_{L0}^{*}U^{*2}}\equiv O(1)\equiv K_{v,L},$$

(32) $$\frac{\lambda_{\mathrm{relax},L}^{*}}{T^{*}}\equiv O(∊)\equiv \lambda_{\mathrm{relax},L}∊,s$$

where $T^{*}$ is the typical period of the wave, which can be related to the wavelength $L^{*}$ and wave speed $U^{*}$ as $U^{*}=L^{*}/T^{*}$.

The nondimensionalization of the energy Eq. (22) is as in Refs. [94], [10]:

(33) $$\frac{3(\kappa -1)\lambda_{G}^{*}}{p_{G0}^{*}\omega^{*}R_{0}^{*}}\frac{5}{4}\frac{T_{0}^{*}}{R_{0}^{*}}=\lambda_{G}∊,$$

where $\lambda_{G}$ is the dimensionless constant of O(1).

### 2.4 Multiple scale analysis

The independent variables $t^{*},x^{*},r^{*},y^{*}$, and $z^{*}$ are nondimensionalized as

(34) $$t=\frac{t^{*}}{T^{*}},\;x=\frac{x^{*}}{L^{*}},\;r=\frac{r^{*}}{L^{*}},\;y=\frac{y^{*}}{L^{*}},\;z=\frac{z^{*}}{L^{*}}\text{.}$$

Next, *t* and *x* are extended to the near and far fields using the dimensionless wave amplitude ∊: [121]

(35) $$t_{0}=t,\;x_{0}=x\;(\text{near field}),$$

(36) $$t_{1}=∊t,\;x_{1}=∊x\;(\text{far field})\text{.}$$

By the assumption of weakly diffracted waves in Eq. (2), the dependence of unknown variables on the radial direction (r,y,z) is smaller than that on the propagative direction *x* [88], [90], [93], [89]. Then, the spatial coordinates r,y, and *z* are defined in the far field only as

(37) $$r_{1/2}=\sqrt{∊}\Gamma_{r}r,\;y_{1/2}=\sqrt{∊}\Gamma_{y}y,\;z_{1/2}=\sqrt{∊}\Gamma_{z}z\;(\text{far field})\text{.}$$

For Eq. (37), the following relationships are introduced: [88], [90], [93], [89]

(38) $$\begin{array}{cc} & r=\frac{a_{r}^{*}}{L^{*}}\frac{r^{*}}{a_{r}^{*}}=\frac{r_{1/2}}{\sqrt{∊}\Gamma_{r}}\;\left(r_{1/2}\equiv \frac{r^{*}}{a_{r}^{*}}\right),\\ & y=\frac{a_{y}^{*}}{L^{*}}\frac{y^{*}}{a_{y}^{*}}=\frac{y_{1/2}}{\sqrt{∊}\Gamma_{y}}\;\left(y_{1/2}\equiv \frac{y^{*}}{a_{y}^{*}}\right),\\ & z=\frac{a_{z}^{*}}{L^{*}}\frac{z^{*}}{a_{z}^{*}}=\frac{z_{1/2}}{\sqrt{∊}\Gamma_{z}}\;\left(z_{1/2}\equiv \frac{z^{*}}{a_{z}^{*}}\right)\text{.} \end{array}$$

All the unknown variables can be regarded as functions of the extended independent variables of Eqs. (35), (36), (37), (38). The derivative operators are expanded as follows:[121]

(39) $$\frac{\partial }{\partial t}=\frac{\partial }{\partial t_{0}}+∊\frac{\partial }{\partial t_{1}},$$

(40) $$\frac{\partial }{\partial x}=\frac{\partial }{\partial x_{0}}+∊\frac{\partial }{\partial x_{1}},$$

(41) $$\frac{\partial }{\partial r}=\sqrt{∊}\Gamma_{r}\frac{\partial }{\partial r_{1/2}},\;\frac{\partial }{\partial y}=\sqrt{∊}\Gamma_{y}\frac{\partial }{\partial y_{1/2}},\;\frac{\partial }{\partial z}=\sqrt{∊}\Gamma_{z}\frac{\partial }{\partial z_{1/2}}\text{.}$$

The unknown variables are expanded as a power series of ∊:

(42) $$\frac{R^{*}}{R_{0}^{*}}=1+∊R_{1}+{∊}^{2}R_{2}+O({∊}^{3}),$$

(43) $$\frac{T_{G}^{*}}{T_{0}^{*}}=1+∊T_{G1}+{∊}^{2}T_{G2}+O({∊}^{3}),$$

(44) $$\frac{p_{L}^{*}}{\rho_{L0}^{*}U^{*2}}=p_{L0}+∊p_{L1}+{∊}^{2}p_{L2}+O({∊}^{3}),$$

(45) $$\frac{q_{L}^{*}}{\rho_{L0}^{*}U^{*2}}=q_{L0}+∊q_{L1}+{∊}^{2}q_{L2}+O({∊}^{3}),$$

(46) $$\frac{u_{x}^{*}}{U^{*}}=∊u_{x1}+{∊}^{2}u_{x2}+O({∊}^{3}),$$

(47) $$\frac{u_{r}^{*}}{U^{*}}={∊}^{3/2}u_{r1}+{∊}^{5/2}u_{r2}+O({∊}^{7/2}),$$

(48) $$\frac{u_{y}^{*}}{U^{*}}={∊}^{3/2}u_{y1}+{∊}^{5/2}u_{y2}+O({∊}^{7/2}),$$

(49) $$\frac{u_{z}^{*}}{U^{*}}={∊}^{3/2}u_{z1}+{∊}^{5/2}u_{z2}+O({∊}^{7/2}),$$

where $u_{x}^{*},u_{r}^{*},u_{y}^{*}$, and $u_{z}^{*}$ are the components of velocity vector $u^{*}$ in the $x^{*},r^{*},y^{*}$, and $z^{*}$ directions, respectively. The magnitudes of $u_{r}^{*},u_{y}^{*}$, and $u_{z}^{*}$ are assumed to be smaller than that of the $u_{x}^{*}$ direction [88], [90], [93], [89], [10]. Then, the expansions of $u_{r}^{*},u_{y}^{*}$, and $u_{z}^{*}$ begin with a higher order than that of $u_{x}^{*}$ in Eqs. (46), (47), (48), (49). The components of displacement vector $d^{*}$ are expanded in the same manner as $u^{*}$:

(50) $$\frac{d_{x}^{*}}{L^{*}}=∊d_{x1}+{∊}^{2}d_{x2}+O({∊}^{3}),$$

(51) $$\frac{d_{r}^{*}}{L^{*}}={∊}^{3/2}d_{r1}+{∊}^{5/2}d_{r2}+O({∊}^{7/2}),$$

(52) $$\frac{d_{y}^{*}}{L^{*}}={∊}^{3/2}d_{y1}+{∊}^{5/2}d_{y2}+O({∊}^{7/2}),$$

(53) $$\frac{d_{z}^{*}}{L^{*}}={∊}^{3/2}d_{z1}+{∊}^{5/2}d_{z2}+O({∊}^{7/2})\text{.}$$

The components of the deviatoric stress tensor $\tau^{*}$ are expanded as

(54) $$\frac{\tau_{\mathrm{xx}}^{*}}{\rho_{L0}^{*}U^{*2}}=∊\tau_{\mathrm{xx}1}+{∊}^{2}\tau_{\mathrm{xx}2}+O({∊}^{3}),$$

(55) $$\frac{\tau_{\mathrm{xr}}^{*}}{\rho_{L0}^{*}U^{*2}}={∊}^{3/2}\tau_{\mathrm{xr}1}+{∊}^{5/2}\tau_{\mathrm{xr}2}+O({∊}^{7/2}),$$

(56) $$\frac{\tau_{\mathrm{xy}}^{*}}{\rho_{L0}^{*}U^{*2}}={∊}^{3/2}\tau_{\mathrm{xy}1}+{∊}^{5/2}\tau_{\mathrm{xy}2}+O({∊}^{7/2}),$$

(57) $$\frac{\tau_{\mathrm{xz}}^{*}}{\rho_{L0}^{*}U^{*2}}={∊}^{3/2}\tau_{\mathrm{xz}1}+{∊}^{5/2}\tau_{\mathrm{xz}2}+O({∊}^{7/2}),$$

where other components that do not affect the result are omitted.

The gas density is assumed to be significantly smaller than the liquid density in the initial state:

(58) $$\frac{\rho_{G0}^{*}}{\rho_{L0}^{*}}\equiv O({∊}^{3})\text{.}$$

The dimensionless liquid pressure is defined as

(59) $$p_{L0}=\frac{p_{L0}^{*}}{\rho_{L0}^{*}U^{*2}}\equiv O(1)\text{.}$$

The liquid density is expanded as [92]

(60) $$\frac{\rho_{L}^{*}}{\rho_{L0}^{*}}=1+{∊}^{2}\rho_{L1}+{∊}^{3}\rho_{L2}+O({∊}^{4}),$$

where the first order of the expansion is determined using Eqs. (23), (27), (44).

To incorporate the weak nonuniformity of the spatial distribution of bubbles in the initial state, void fraction α is expanded as [88], [90], [93], [89], [10]

(61) $$\frac{\alpha }{\alpha_{0}}=1+∊[\alpha_{1}+\delta (x_{1})]+{∊}^{2}\alpha_{2}+O({∊}^{3}),$$

where δ is the known variable that represents the spatial nonuniformity of the void fraction in the initial state. The effect of the spatial nonuniformity of the void fraction is assumed to appear only in the far field; then, δ is the function of $x_{1}$ only [88], [90], [93], [89], [10].

## 3 Results of theoretical analysis

The scale parameters of Eqs. (27), (28), (29), (30), (31), (32), (33), derivative operators of Eq. (39), and expansions of the unknown variables of Eqs. (42), (43), (44), (45), (46), (47), (48), (49), (50), (51), (52), (53), (54), (55), (56), (57), (58), (59), (60), (61) are substituted into basic Eqs. (11), (12), (13), (14), (15), (16), (17), (18), (19), (20), (21), (22), (23), (24).

### 3.1 Approximation of first order

From the approximations of O(∊), linear equations are obtained for Eqs. (11), (12), (13), as follows.

(62) $$\frac{\partial \alpha_{1}}{\partial t_{0}}-3\frac{\partial R_{1}}{\partial t_{0}}+\frac{\partial u_{x1}}{\partial x_{0}}=0,$$

(63) $$\alpha_{0}\frac{\partial \alpha_{1}}{\partial t_{0}}-\left(1-\alpha_{0}\right)\frac{\partial u_{x1}}{\partial x_{0}}=0,$$

(64) $$\left(1-\alpha_{0}\right)\frac{\partial u_{x1}}{\partial t_{0}}+\frac{\partial p_{L1}}{\partial x_{0}}-\frac{\partial \tau_{\mathrm{xx}1}}{\partial x_{0}}-(K_{e,L}-\frac{2}{3}G_{L})\frac{\partial^{2}d_{x1}}{\partial x_{0}^{2}}=0,$$

(65) $$\frac{\partial T_{G1}}{\partial t_{0}}+3(\kappa -1)\frac{\partial R_{1}}{\partial t_{0}}=0,$$

(66) $$p_{G0}T_{G1}-p_{L1}+3\left(\kappa -1\right)p_{G0}R_{1}-\frac{\Delta^{2}}{\Omega^{2}}R_{1}=0,$$

(67) $$\tau_{\mathrm{xx}1}-2G_{L}\frac{\partial d_{x1}}{\partial x_{0}}=0\text{.}$$

The velocity and displacement are related by Eq. (14) as

(68) $$u_{x1}=\frac{\partial d_{x1}}{\partial t_{0}}\text{.}$$

From the approximation of the Keller–Miksis Eq. (17), the linear natural frequency of bubble oscillation $\omega_{B}^{*}$ is obtained:

(69) $$\omega_{B}^{*}=\sqrt{\frac{3\kappa p_{G0}^{*}-2\sigma^{*}/R_{0}^{*}+4G_{L}^{*}}{\rho_{L0}^{*}R_{0}^{*2}}}\text{.}$$

In Eq. (69), the effect of liquid elasticity at the bubble–liquid interface [91] is introduced to our previous works [88], [94], [95], [93]. Note that $\omega_{B}^{*}$ of Eq. (69) is obtained from the linear approximation, then the actual frequency of bubble oscillation will be shifted by accumulation of nonlinearity and wave amplitude [122], [123]. In addition, bubble–bubble interaction [104], [105], [106], [46], [44], [39] and dual frequency ultrasound [129], [130], [131] also affect the frequency of bubble oscillation, however these are not considered in this study.

Eqs. (62), (63), (64), (65), (66), (67) are combined into a single equation of $R_{1}$ as

(70) $$\frac{\partial^{2}R_{1}}{\partial t_{0}^{2}}-v_{P}^{2}\frac{\partial^{2}R_{1}}{\partial x_{0}^{2}}=0,$$

where the phase velocity $v_{P}$ is given by

(71) $$v_{P}=\sqrt{\frac{\Delta^{2}/\Omega^{2}+3\alpha_{0}\left(K_{e,L}+4G_{L}/3\right)}{3\alpha_{0}\left(1-\alpha_{0}\right)}}\text{.}$$

For simplicity, $v_{P}\equiv 1$ is imposed and the typical wave speed $U^{*}$ is given by

(72) $$U^{*}=\sqrt{\frac{\omega_{B}^{*2}R_{0}^{*2}\rho_{L0}^{*}+3\alpha_{0}\left(K_{e,L}^{*}+4G_{L}^{*}/3\right)}{3\alpha_{0}\left(1-\alpha_{0}\right)\rho_{L0}^{*}}}\text{.}$$

In Eq. (72), the effect of the elasticity of the entire liquid is newly introduced to our previous works [88], [94], [95], [93].

Next, the variable transformation is introduced as

(73) $$\phi_{0}=t_{0}-x_{0}\text{.}$$

Then, the equation describing the right-running waves is obtained.

(74) $$\frac{\partial f}{\partial t_{0}}+\frac{\partial f}{\partial x_{0}}=0\text{.}$$

The variable transformation of Eq. (73) is introduced into approximated Eqs. (62), (63), (64), (65), (66), (67), (68)), and all unknown variables are written in terms of $R_{1}=f$ as follows.

(75) $$\begin{array}{cc} & \alpha_{1}=s_{1}f,\;u_{x1}=s_{2}f,\;T_{G1}=s_{3}f,\\ & p_{L1}=s_{4}f,\;d_{x1}=s_{5}\int f\;d\phi_{0},\;\tau_{\mathrm{xx}1}=s_{6}f, \end{array}$$

where

(76) $$s_{1}=3\left(1-\alpha_{0}\right),$$

(77) $$s_{2}=-3\alpha_{0},$$

(78) $$s_{3}=-3\left(\kappa -1\right),$$

(79) $$s_{4}=3\alpha_{0}\left(K_{e,L}+\frac{4}{3}G_{L}\right)-3\alpha_{0}\left(1-\alpha_{0}\right),$$

(80) $$s_{5}=-3\alpha_{0},$$

(81) $$s_{6}=6\alpha_{0}G_{L}\text{.}$$

### 3.2 Approximation of radial direction

From the approximations of $O({∊}^{2/3})$, the radial components of the momentum conservation Eq. (13) are

(82) $$\left(1-\alpha_{0}\right)\frac{\partial u_{r1}}{\partial t_{0}}+\Gamma_{r}\frac{\partial p_{L1}}{\partial r_{1/2}}-\frac{\partial \tau_{\mathrm{xr}1}}{\partial x_{0}}-\left(K_{e,L}-\frac{2}{3}G_{L}\right)\Gamma_{r}\frac{\partial^{2}d_{x1}}{\partial x_{0}\partial r_{1/2}}=0,$$

(83) $$\left(1-\alpha_{0}\right)\frac{\partial u_{y1}}{\partial t_{0}}+\Gamma_{y}\frac{\partial p_{L1}}{\partial y_{1/2}}-\frac{\partial \tau_{\mathrm{xy}1}}{\partial x_{0}}-\left(K_{e,L}-\frac{2}{3}G_{L}\right)\Gamma_{y}\frac{\partial^{2}d_{x1}}{\partial x_{0}\partial y_{1/2}}=0,$$

(84) $$\left(1-\alpha_{0}\right)\frac{\partial u_{z1}}{\partial t_{0}}+\Gamma_{z}\frac{\partial p_{L1}}{\partial z_{1/2}}-\frac{\partial \tau_{\mathrm{xz}1}}{\partial x_{0}}-\left(K_{e,L}-\frac{2}{3}G_{L}\right)\Gamma_{z}\frac{\partial^{2}d_{x1}}{\partial x_{0}\partial z_{1/2}}=0\text{.}$$

The components of the deviatoric stress tensor (16) related to the radial directions are

(85) $$\tau_{\mathrm{xr}1}-G_{L}\frac{\partial d_{r1}}{\partial x_{0}}-G_{L}\Gamma_{r}\frac{\partial d_{x1}}{\partial r_{1/2}}=0,$$

(86) $$\tau_{\mathrm{xy}1}-G_{L}\frac{\partial d_{y1}}{\partial x_{0}}-G_{L}\Gamma_{y}\frac{\partial d_{x1}}{\partial y_{1/2}}=0,$$

(87) $$\tau_{\mathrm{xz}1}-G_{L}\frac{\partial d_{z1}}{\partial x_{0}}-G_{L}\Gamma_{z}\frac{\partial d_{x1}}{\partial z_{1/2}}=0\text{.}$$

The velocity and displacement are related by Eq. (14) as

(88) $$u_{r1}=\frac{\partial d_{r1}}{\partial t_{0}},\;u_{y1}=\frac{\partial d_{y1}}{\partial t_{0}},\;u_{z1}=\frac{\partial d_{z1}}{\partial t_{0}}\text{.}$$

The variable transformation Eq. (73) is introduced into Eqs. (82), (83), (84), (85), (86), (87), (88):

(89) $$\frac{\partial u_{r1}}{\partial \phi_{0}}=3\alpha_{0}\Gamma_{r}\frac{\partial f}{\partial r_{1/2}},$$

(90) $$\frac{\partial u_{y1}}{\partial \phi_{0}}=3\alpha_{0}\Gamma_{y}\frac{\partial f}{\partial y_{1/2}},$$

(91) $$\frac{\partial u_{z1}}{\partial \phi_{0}}=3\alpha_{0}\Gamma_{z}\frac{\partial f}{\partial z_{1/2}},$$

where the forms of (89)–(91) are the same as in our previous work [93].

### 3.3 Approximation of second order

From the approximations of $O({∊}^{2})$, the following equations are obtained for Eqs. (11), (12), (13) as

(92) $$\frac{\partial \alpha_{2}}{\partial t_{0}}-3\frac{\partial R_{2}}{\partial t_{0}}+\frac{\partial u_{x2}}{\partial x_{0}}=K_{1},$$

(93) $$\alpha_{0}\frac{\partial \alpha_{2}}{\partial t_{0}}-\left(1-\alpha_{0}\right)\frac{\partial u_{x2}}{\partial x_{0}}=K_{2},$$

(94) $$\left(1-\alpha_{0}\right)\frac{\partial u_{x2}}{\partial t_{0}}+\frac{\partial p_{L2}}{\partial x_{0}}-\frac{\partial \tau_{\mathrm{xx}2}}{\partial x_{0}}-\left(K_{e,L}-\frac{2}{3}G_{L}\right)\frac{\partial^{2}d_{x2}}{\partial x_{0}^{2}}=K_{3},$$

(95) $$\frac{\partial T_{G2}}{\partial t_{0}}+3\left(\kappa -1\right)\frac{\partial R_{2}}{\partial t_{0}}=K_{4},$$

(96) $$p_{G0}T_{G2}-p_{L2}+3\left(\kappa -1\right)p_{G0}R_{2}-\frac{\Delta^{2}}{\Omega^{2}}R_{2}=K_{5},$$

(97) $$\tau_{\mathrm{xx}2}-2G_{L}\frac{\partial d_{x2}}{\partial x_{0}}=K_{6}\text{.}$$

The explicit forms of the inhomogeneous terms $K_{i}\;(i=1,\ldots ,6)$ are shown in Appendix A. Eqs. (92), (93), (94), (95), (96), (97) are combined into a single equation:

(98) $$\frac{\partial^{2}R_{2}}{\partial t_{0}^{2}}-\frac{\partial^{2}R_{2}}{\partial x_{0}^{2}}=K\text{.}$$

The inhomogeneous term *K* is given by

(99) $$K=-\frac{1}{3}\frac{\partial K_{1}}{\partial t_{0}}+\frac{1}{3\left(1-\alpha_{0}\right)}\left(K_{e,L}+\frac{4}{3}G_{L}\right)\int \frac{\partial^{2}K_{1}}{\partial x_{0}^{2}}{\mathrm{dt}}_{0}+\frac{1}{3\alpha_{0}}\frac{\partial K_{2}}{\partial t_{0}}-\frac{1}{3\alpha_{0}\left(1-\alpha_{0}\right)}\left(K_{e,L}+\frac{4}{3}G_{L}\right)\int \frac{\partial^{2}K_{2}}{\partial x_{0}^{2}}{\mathrm{dt}}_{0}+\frac{1}{3\alpha_{0}(1-\alpha_{0})}\frac{\partial K_{3}}{\partial x_{0}}-\frac{p_{G0}}{3\alpha_{0}(1-\alpha_{0})}\int \frac{\partial^{2}K_{4}}{\partial x_{0}^{2}}dt_{0}+\frac{1}{3\alpha_{0}(1-\alpha_{0})}\frac{\partial^{2}K_{5}}{\partial x_{0}^{2}}+\frac{1}{3\alpha_{0}\left(1-\alpha_{0}\right)}\frac{\partial^{2}K_{6}}{\partial x_{0}^{2}},$$

As the solvable condition for Eq. (98), K=0 is imposed [88], [90], [92], [89], and the following relation is obtained for 2D and 3D spatial cases:

(100) $$2\frac{\partial }{\partial \phi_{0}}\left[\frac{\partial f}{\partial x_{1}}+\frac{\partial f}{\partial t_{1}}+\Pi_{02}\frac{\partial f}{\partial t_{1}}+\Pi_{01}\frac{\partial f}{\partial \phi_{0}}+\Pi_{4}\delta (x_{1})\frac{\partial f}{\partial \phi_{0}}+\Pi_{1}f\frac{\partial f}{\partial \phi_{0}}+\Pi_{21}\frac{\partial^{2}f}{\partial \phi_{0}^{2}}+\Pi_{22}f+\Pi_{3}\frac{\partial^{3}f}{\partial \phi_{0}^{3}}\right]=\left\{\begin{array}{cc} \Gamma_{r}^{2}\left(\frac{1}{r_{1/2}}\frac{\partial f}{\partial r_{1/2}}+\frac{\partial^{2}f}{\partial r_{1/2}^{2}}\right) & (\text{for 2D case}),\\ \Gamma_{y}^{2}\frac{\partial f}{\partial y_{1/2}^{2}}+\Gamma_{z}^{2}\frac{\partial f}{\partial z_{1/2}^{2}} & (\text{for 3D case})\text{.} \end{array}\right)$$

The derivative operators (39)–(41), equation of the near field (74), equations of the radial direction (89)–(91), and Eq. (100) are combined:

(101) $$\frac{\partial }{\partial t}\left\{\frac{\partial f}{\partial x}+\frac{\partial f}{\partial t}+\Pi_{02}\frac{\partial f}{\partial t}+∊\left[\Pi_{01}\frac{\partial f}{\partial t}+\Pi_{4}\delta (x_{1})\frac{\partial f}{\partial t}+\Pi_{1}f\frac{\partial f}{\partial t}+\Pi_{21}\frac{\partial^{2}f}{\partial t^{2}}+\Pi_{22}f+\Pi_{3}\frac{\partial^{3}f}{\partial t^{3}}\right]\right\}=\left\{\begin{array}{cc} \frac{1}{2}\left(\frac{1}{r}\frac{\partial f}{\partial r}+\frac{\partial^{2}f}{\partial r^{2}}\right) & (\mathrm{for}2\mathrm{Dcase}),\\ \frac{1}{2}\left(\frac{\partial^{2}f}{\partial y^{2}}+\frac{\partial^{2}f}{\partial z^{2}}\right) & (\mathrm{for}3\mathrm{Dcase})\text{.} \end{array}\right)$$

Finally, the KZK equation is obtained:

(102) $$\frac{\partial }{\partial \tau }\left(\frac{\partial f}{\partial X}+\Pi_{1}f\frac{\partial f}{\partial \tau }+\Pi_{21}\frac{\partial^{2}f}{\partial \tau^{2}}+\Pi_{22}f+\Pi_{3}\frac{\partial^{3}f}{\partial \tau^{3}}\right)=\Delta_{⊥}f,$$

where $\Delta_{⊥}$ is the Laplacian operator given for 2D and 3D spatial cases by

(103) $$\Delta_{⊥}f=\left\{\begin{array}{cc} \frac{\Gamma_{r}^{2}}{2}\left(\frac{1}{R}\frac{\partial f}{\partial R}+\frac{\partial^{2}f}{\partial R^{2}}\right) & (\mathrm{for}2\mathrm{Dcase}),\\ \frac{1}{2}\left(\Gamma_{y}^{2}\frac{\partial^{2}f}{\partial Y^{2}}+\Gamma_{z}^{2}\frac{\partial^{2}f}{\partial Z^{2}}\right) & (\mathrm{for}3\mathrm{Dcase})\text{.} \end{array}\right)$$

The following variable transformations are used:

(104) $$\tau =t-\left\{1+\Pi_{02}+∊\left[\Pi_{01}+\Pi_{4}\delta \left(x_{1}\right)\right]\right\}x,$$

(105) X=∊x,

(106) $$\left\{\begin{array}{cc} R=\sqrt{∊}\Gamma_{r}r & (\mathrm{for}2\mathrm{Dcase}),\\ Y=\sqrt{∊}\Gamma_{y}y,\;Z=\sqrt{∊}\Gamma_{z}z & (\mathrm{for}3\mathrm{Dcase}), \end{array}\right)$$

where τ is the retarded time. In the KZK Eq. (102), the right-hand side with the Laplacian operator represents the diffraction (focusing) effect.

### 3.4 Coefficients of KZK equation

$\Pi_{01},\Pi_{02}$, and $\Pi_{4}$ are the advection coefficients given by

(107) $$\Pi_{01}={\left[1-\alpha_{0}-\left(K_{e,L}+\frac{4}{3}G_{L}\right)\right]}^{2}\frac{V^{2}}{2},$$

(108) $$\Pi_{02}=-\left(K_{e,L}+\frac{4}{3}G_{L}\right)\frac{1}{2\left(1-\alpha_{0}\right)},$$

(109) $$\Pi_{4}=\frac{1-2\alpha_{0}}{2(1-\alpha_{0})}-\left(K_{e,L}+\frac{4}{3}G_{L}\right)\frac{1}{2(1-\alpha_{0})}\text{.}$$

By the effect of the elasticity of the entire liquid, the new coefficient $\Pi_{02}$ is introduced in this study and terms are added to $\Pi_{01}$ and $\Pi_{4}$ from our previous work [10]. The spatial nonuniformity of the initial bubble distribution $\delta (x_{1})$ only appears in the variable transformation of Eq. (104), in which it only affects the advection term [88], [90], [93], [89]. $\Pi_{1}$ is the nonlinear coefficient given by

(110) $$\Pi_{1}=\frac{1}{6}\left[k_{1}-\frac{1}{1-\alpha_{0}}\left(K_{e,L}+\frac{4}{3}G_{L}\right)k_{1}-\frac{k_{2}}{\alpha_{0}}+\frac{1}{1-\alpha_{0}}\left(K_{e,L}+\frac{4}{3}G_{L}\right)\frac{k_{2}}{\alpha_{0}}+\frac{k_{3}}{\alpha_{0}\left(1-\alpha_{0}\right)}+\frac{p_{G0}k_{4}}{\alpha_{0}\left(1-\alpha_{0}\right)}+\frac{k_{5}}{\alpha_{0}\left(1-\alpha_{0}\right)}+\frac{k_{6}}{\alpha_{0}\left(1-\alpha_{0}\right)}\right],$$

where

(111) $$k_{1}=-6\left(2-s_{1}\right)-2s_{2}\left(3-s_{1}\right),$$

(112) $$k_{2}=2\alpha_{0}s_{1}s_{2},$$

(113) $$k_{3}=0,$$

(114) $$k_{4}=-3\left(\kappa -1\right)\left(3\kappa -4+2s_{3}\right),$$

(115) $$k_{5}=\left[8G_{L}-6\left(\kappa -2\right)p_{G0}+2\frac{\Delta^{2}}{\Omega^{2}}\right]-6p_{G0}s_{3},$$

(116) $$k_{6}=0\text{.}$$

$\Pi_{21}$ is the dissipation coefficient divided into five terms for each factor and region:

(117) Π21=ΠC︸liquidcompressibility+Πv,E︸viscosityofentireliquid+Πv,I︸liquidviscosityofbubble--liquidinterface+Πe,E︸elasticityofentireliquid+Πe,I︸liquidelasticityofbubble--liquidinterface,

where

(118) $$\Pi_{C}=-\frac{V\Delta }{2}<0,$$

(119) $$\Pi_{v,E}=-\frac{2\mu_{L}}{3\left(1-\alpha_{0}\right)}<0,$$

(120) $$\Pi_{v,I}=-\frac{2\mu_{L}}{3\alpha_{0}\left(1-\alpha_{0}\right)}<0,$$

(121) $$\Pi_{e,E}=\frac{V\Delta }{2\left(1-\alpha_{0}\right)}\left(K_{e,L}+\frac{4}{3}G_{L}\right)+\frac{\lambda_{\mathrm{relax},L}G_{L}}{\left(1-\alpha_{0}\right)}>0,$$

(122) $$\Pi_{e,I}=\frac{2\lambda_{\mathrm{relax},L}G_{L}}{3\alpha_{0}\left(1-\alpha_{0}\right)}>0\text{.}$$

$\Pi_{22}$ and $\Pi_{3}$ are the dissipation and dispersion coefficients, respectively, given by

(123) $$\Pi_{22}=\frac{p_{G0}\left(\kappa -1\right)}{2\alpha_{0}\left(1-\alpha_{0}\right)}\lambda_{G},$$

(124) $$\Pi_{3}=-\frac{\Delta^{2}}{6\alpha_{0}\left(1-\alpha_{0}\right)}\text{.}$$

Whereas $\Pi_{21}$ depends on the compressibility, viscosity, and elasticity of the liquid phase, $\Pi_{22}$ depends on the thermal conductivity of the gas phase.

### 3.5 Effects of elasticity

**Fig. 3:**
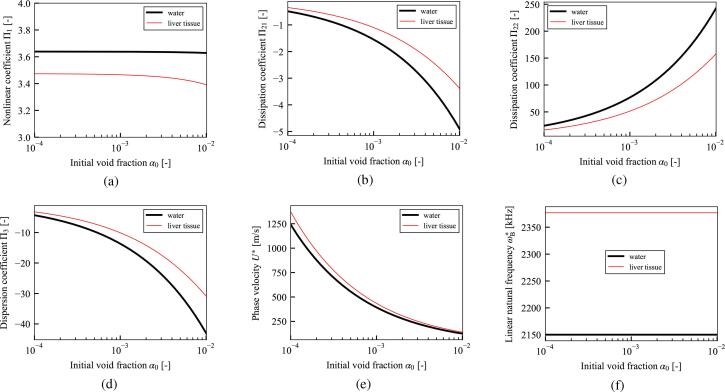
(a) Nonlinear coefficient $\Pi_{1}$, (b) and (c) dissipation coefficients $\Pi_{21}$ and $\Pi_{22}$, (d) dispersion coefficient $\Pi_{3}$, (e) phase velocity $U^{*}$, and (f) linear natural frequency of bubble oscillation $\omega_{B}^{*}$, versus initial void fraction $\alpha_{0}$. The black and red lines indicate cases in which the liquid phase is water and liver tissue, respectively. The material properties of water and liver tissue are shown in Table 1. Parameters: initial bubble radius $R_{0}^{*}=10\;\mu m$, frequency of sound source $f^{*}=100$ kHz, pressure amplitude of sound source $p_{0}^{*}=100$ kPa, initial liquid pressure $p_{L0}^{*}=101325$ Pa, and initial temperature of gas and liquid phases $T_{0}^{*}=36.0{\;}^{\circ }$C. The gas inside the bubble is air with the ratio of specific heat κ=1.4 and thermal conductivity $\lambda_{G}^{*}=0.025$ W/(m·K).

Fig. 3 shows the coefficients for cases in which the liquid phase is water (black) and liver tissue (red). The properties of the water and liver tissue used in the calculations are shown in Table 1. Unless otherwise stated, these values are used in all calculations in Sections 3.5, 3.6, 3.7, 4. As shown in Fig. 3, elasticity decreases the magnitudes of the nonlinear coefficient $\Pi_{1}$, dissipation coefficients $\Pi_{21}$ and $\Pi_{22}$, and dispersion coefficient $\Pi_{3}$, and increases the phase velocity $U^{*}$ and linear natural frequency of bubble oscillation $\omega_{B}^{*}$. Decrease of nonlinearity by the liquid elasticity is qualitatively consistent with the previous result [34] focusing of single bubble behavior.

**Table 1:** Material properties of liquid phase.

	Water	Liver tissue [47], [128]
Rigidity $G_{L}^{*}$ [kPa]	0	40
Poisson’s ratio $\nu_{L}$ [-]	-	0.45
Relaxation time $\lambda_{\mathrm{relax},L}^{*}$ [s]	0	3.0 ×$10^{-9}$
Viscosity $\mu_{L}^{*}$ [Pa·s]	0.001	0.001
Surface tension $\sigma^{*}$ [N/m]	0.056	0.056
Density $\rho_{L0}^{*}$ [kg/m^3^]	998	1100
Wave speed $c_{L0}^{*}$ [m/s]	1486	1549

### 3.6 Comparison of three viscoelastic models

**Fig. 4:**
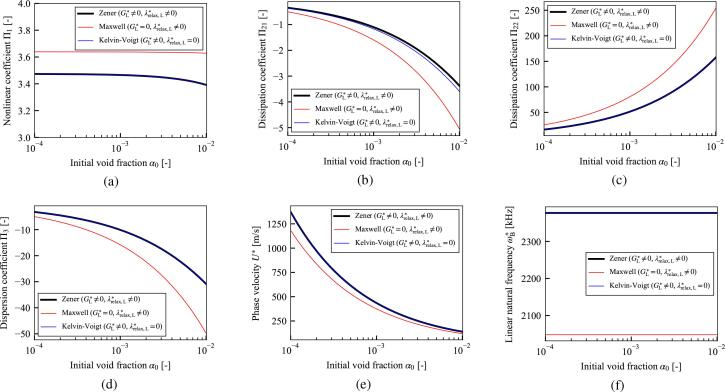
The same parameters as in Fig. 3 are shown for the case of liver tissue. The black line represents the case in which the viscoelastic model is the Zener model ($G_{L}^{*}\;\neq \;0,\lambda_{\mathrm{relax},L}^{*}\;\neq \;0$), the red line represents the case of the Maxwell model ($G_{L}^{*}=0,\lambda_{\mathrm{relax},L}^{*}\;\neq \;0$), and the blue line represents the case of Kelvin–Voigt model ($G_{L}^{*}\;\neq \;0,\lambda_{\mathrm{relax},L}^{*}=0$). The other conditions are same as in Fig. 3.

In Fig. 4, the three viscoelastic models; Zener model, Maxwell model, and Kelvin–Voigt model are compared. The Zener model, that are the rigidity $G_{L}^{*}\;\neq \;0$ and the relaxation time $\lambda_{\mathrm{relax},L}^{*}\;\neq \;0$, corresponds to the generalization of the Maxwell model ($G_{L}^{*}=0$) and the Kelvin–Voigt model ($\lambda_{\mathrm{relax},L}^{*}=0$). From the Fig. 4b, the magnitude of the dissipation coefficient $\Pi_{21}$ is decreased in case of the Zener model compare to the Kelvin–Voigt model. Then, the relaxation time of the liquid phase decrease the dissipation of the ultrasound. This result is qualitatively consistent with the result for the case of single bubble by Warnez and Johnsen [42] that incorporating the relaxation time increases bubble growth. However, all parameters shown in Fig. 4 are almost affected by the generalization from the Maxwell model to the Zener model. Hence, the effect of rigidity $G_{L}^{*}$ is quite larger than that of the relaxation time $\lambda_{\mathrm{relax},L}^{*}$ for all parameters.

### 3.7 Comparison of elasticity of entire liquid and bubble–liquid interface

**Fig. 5:**
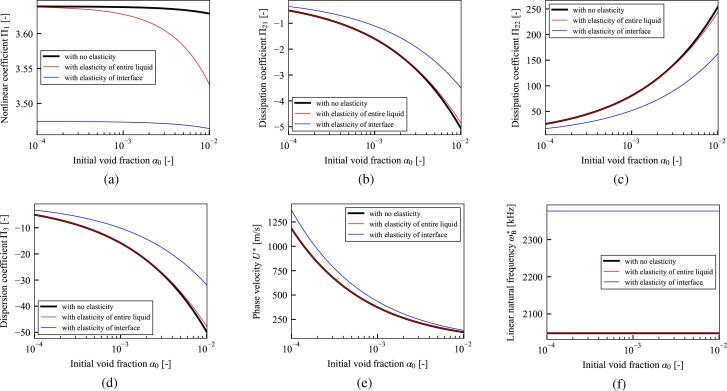
The same parameters as in Fig. 3 are shown for the case of liver tissue. The black line represents the case in which both elasticities are not considered. The red line represents the case in which only the elasticity of the entire liquid is considered, and the blue line represents the case in which only the elasticity of the bubble–liquid interface is considered. The other conditions are same as in Fig. 3.

**Fig. 6:**
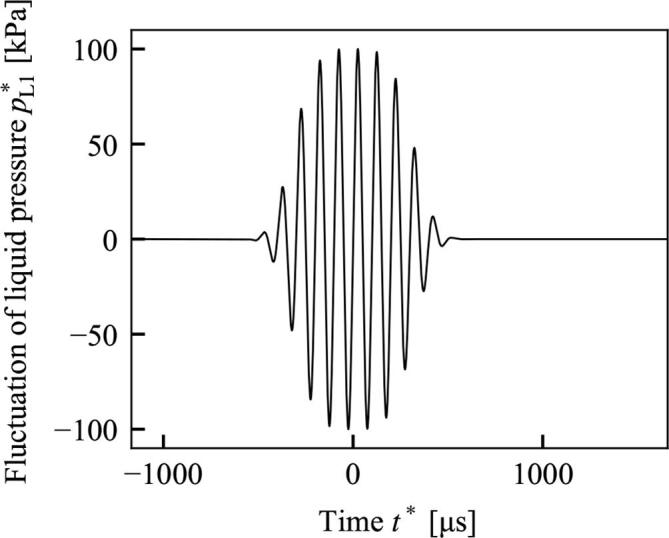
Boundary condition for calculating Fig. 7, Fig. 8 at the center of the sound source ($x^{*}=0\;\mathrm{mm}$ and $r^{*}=0\;\mathrm{mm}$).

In Fig. 5, the effects of the liquid elasticity of the entire liquid and bubble–liquid interface are compared. As the effects of each factor become larger, the red (elasticity of entire liquid) and blue (elasticity of bubble–liquid interface) lines differ from the black line (both elasticities not considered). (See Fig. 6).

As shown in Fig. 5, the nonlinear coefficient $\Pi_{1}$ is decreased by both elasticities; the effect of the bubble–liquid interface is large at the wide range of the initial void fraction $\alpha_{0}$, while the effect of the entire liquid gradually increases as the value of $\alpha_{0}$ increases. The magnitudes of the dissipation coefficients $\Pi_{21}$ and $\Pi_{22}$ and the dispersion coefficient $\Pi_{3}$ are affected by the elasticity of the bubble–liquid interface, whereas the effect of the elasticity of the entire liquid is only slight and increases with the initial void fraction $\alpha_{0}$. The phase velocity $U^{*}$ and linear natural frequency of the bubble oscillation $\omega_{B}^{*}$ increase when the elasticity of the bubble–liquid interface is considered, whereas the effects of the elasticity of the entire liquid are not observed.

### 3.8 Limitation of KZK Eq. (102)

The resultant KZK Eq. (102) includes the nonlinear term with coefficient $\Pi_{1}$ owing to nonlinearity of ultrasound propagation, while the dissipation and dispersion terms are limited to linear form. However, effects of nonlinear dissipation [124], [125], [126], [127] will become large particularly in case of high amplitude. Two main methods to incorporate the nonlinear dissipation are (i) extending to over third order analysis from the second order of this study, (ii) nondimensionalization with lower order of ∊(≪1) for liquid viscosity of Eq. (29). These extensions will significantly change the framework of theoretical analysis and the resultant equation will be no longer the form of original KZK equation, but be very important for practial applications then be presented in our future work.

## 4 Numerical example

### 4.1 Method

The KZK equation of the spatial 2D form of Eq. (102) is numerically solved. As in our previous work [10], the finite-difference time-domain scheme [58], [57] is used, which has been widely used to simulate focused ultrasound in single phase liquid [57], [58], [59], [60], [61], [62], [63], [64], [65], [66], [67], [68]. To solve the fluctuation of liquid pressure $p_{L1}$, the KZK Eq. (102) is rewritten using the relation of the fluctuation of bubble radius *f* and liquid pressure $p_{L1}$ in Eq. (75):

(125) $$\begin{array}{cc} & \frac{\partial p_{L1}}{\partial X}=\frac{1}{4G_{r}}\int_{-\infty }^{\tau }\left(\frac{\partial^{2}p_{L1}}{\partial R^{2}}+\frac{1}{R}\frac{\partial p_{L1}}{\partial R}\right)d\tau \\ & -\frac{\Pi_{1}}{s_{4}}p_{L1}\frac{\partial p_{L1}}{\partial \tau }-\Pi_{21}\frac{\partial^{2}p_{L1}}{\partial \tau^{2}}-\Pi_{22}p_{L1}-\Pi_{3}\frac{\partial^{3}p_{L1}}{\partial \tau^{3}}, \end{array}$$

where the focusing gain $G_{r}$ is given by

(126) $$G_{r}=\frac{\omega^{*}a_{r}^{*2}}{2U^{*}d_{f}^{*}}\text{.}$$

The variable transformations (104) are rewritten again as

(127) $$\tau =\omega^{*}\left(t^{*}-\left\{1+\Pi_{02}+∊\left[\Pi_{01}+\Pi_{4}\delta \left(x_{1}\right)\right]\right\}\frac{x^{*}}{U^{*}}\right),$$

(128) $$X=\frac{x^{*}}{d_{f}^{*}},$$

(129) $$R=\frac{r^{*}}{a_{r}^{*}}\text{.}$$

The step sizes of each direction are Δτ=2π/240,ΔX=0.00025, and ΔR=0.01, and the calculation regions are $\tau_{\min}≦\tau ≦\tau_{\max},0≦X≦X_{\max}$, and $0≦R≦R_{\max}$. To suppress the numerical oscillation, the calculation regions need to be sufficiently large for the direction of τ and *R*; then, $\tau_{\min}=-(G_{r}+24\pi ),\tau_{\max}=34\pi$, and $R_{\max}=400$ are set.

Next, the KZK Eq. (125) is split into three parts and solved term by term:

(130) $$\frac{\partial p_{L1}}{\partial X}=\frac{1}{4G_{r}}\int_{-\infty }^{\tau }\left(\frac{\partial^{2}p_{L1}}{\partial R^{2}}+\frac{1}{R}\frac{\partial p_{L1}}{\partial R}\right)d\tau ,$$

(131) $$\frac{\partial p_{L1}}{\partial X}=-\Pi_{21}\frac{\partial^{2}p_{L1}}{\partial \tau^{2}}-\Pi_{22}p_{L1}-\Pi_{3}\frac{\partial^{3}p_{L1}}{\partial \tau^{3}},$$

(132) $$\frac{\partial p_{L1}}{\partial X}=-\frac{\Pi_{1}}{s_{4}}p_{L1}\frac{\partial p_{L1}}{\partial \tau }\text{.}$$

The diffraction term of Eq. (130) is solved by the implicit backward finite difference (IBFD) method in the *R* direction with the truncation error of order $\Delta X+{\left(\Delta \tau \right)}^{2}+\Delta R$. The dissipation and dispersion terms of Eq. (131) are also solved using the IBFD method in the τ direction. The second and third derivative terms of Eq. (131) are discretized using the central difference method [10], [58], [57]. The truncation error for solving Eq. (131) is of order ${\left(\Delta X\right)}^{2}+{\left(\Delta \tau \right)}^{2}$. The nonlinear term of Eq. (132) is solved using the implicit analytical solution with an error of order ${\left(\Delta \tau \right)}^{2}$. In addition, the error caused by separately introducing the diffraction, dissipation, dispersion, and nonlinear effects is of order ${\left(\Delta X\right)}^{2}$. The total error is estimated as $\Delta X+{\left(\Delta \tau \right)}^{2}+\Delta R$ [58], [57].

The boundary condition at X=0 for the focused sound source is given by

(133) $$\begin{array}{cc} & p_{L1}(X=0,\;\tau ,\;R)\\ & =\left\{\begin{array}{cc} \exp\left\{-{\left[\frac{2(\tau +G_{r}R^{2})}{T_{d}}\right]}^{m}\right\}\frac{p_{0}^{*}}{∊\rho_{L0}^{*}U^{*2}}\sin(\tau +G_{r}R^{2}) & (0≦R≦1),\\ 0 & (1<R), \end{array}\right) \end{array}$$

where $p_{0}^{*}$ is the amplitude of the source pressure. The wave is only given on the sound source, 0≦R≦1. As shown in Fig. 5, the wave at the sound source ($x^{*}=0\;\mathrm{mm}$ and $r^{*}=0\;\mathrm{mm}$) is amplitude-modulated by the form of the exponential functuion as in Eq. (133). In Eq. (133), $T_{d}$ and *m* are the effective duration and the rise time of pulses, respecrively [57], [58], [60], and set as $T_{d}=14\pi$ and m=4 to radiate approximately 10 pulses.

### 4.2 Results

**Fig. 7:**
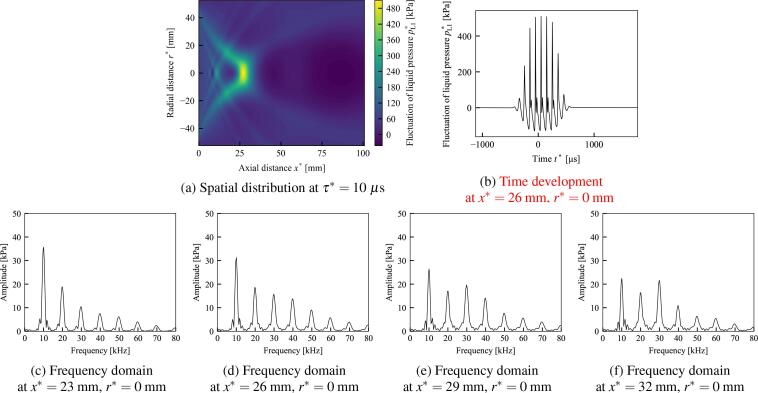
The case in which the liquid is water and the initial void fraction is $\alpha_{0}=0.00025$. (a) is the spatial distributions of the dimensional fluctuation of the liquid pressure $p_{L1}^{*}$ at the retarded time $\tau^{*}=10\;\mu s$. (b) is the time development at $x^{*}=26\;\mathrm{mm}$ and $r^{*}=0\;\mathrm{mm}$ of (a). (c)(d)(e) and (f) are the frequency domains at each point of (a) obtained using the fast Fourier transform. Parameters: initial bubble radius $R_{0}^{*}=100\;\mu m$, frequency of sound source $f^{*}=10$ kHz, pressure amplitude of sound source $p_{0}^{*}=100$ kPa, radius of sound source $a_{r}^{*}=50$ mm, and geometric focal length $d_{f}^{*}=100$ mm.

**Fig. 8:**
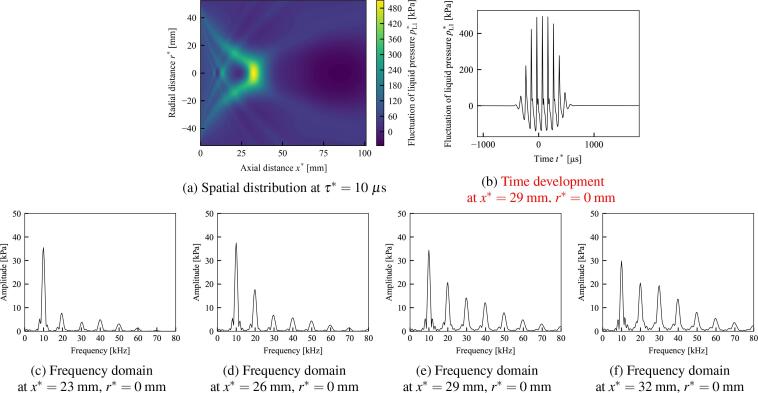
The case in which the liquid is liver tissue and the initial void fraction is $\alpha_{0}=0.0005$. All other conditions are the same as in Fig. 7.

In Fig. 7, Fig. 8, the spatial distribution of the first-order dimensional fluctuation of the liquid pressure $p_{L1}^{*}=\rho_{L0}^{*}U^{*}∊p_{L1}$ at a certain retarded time $\tau^{*}$, the time development of the liquid pressure $p_{L1}^{*}$ at a certain point, and the frequency domain of the time development obtained using FFT are shown. The wave distortion of the time development of $p_{L1}^{*}$ in Fig. 7, Fig. 8, the generation of higher harmonics in Figs. 7–8f are due to the nonlinearity. In Fig. 7, Fig. 8, the points with the maximum value of $p_{L1}^{*}$ are approximately $x^{*}=26\;\mathrm{mm}$ and $x^{*}=29\;\mathrm{mm}$, respectively, and are near the sound source of the geometric focal point at $x^{*}=100\;\mathrm{mm}$. In Table 2, the parameters and resultant values of the calculation are shown for five different initial void fractions $\alpha_{0}$, including the cases shown in Fig. 7, Fig. 8. Because of the Assumption (a-5) in Section 2.1, sufficiently small value of initial void fraction is used. In $\alpha_{0}=0.0001$, the maximum value of $p_{L1}^{*}$ in the case of water exceeds that of liver tissue. However, in $\alpha_{0}⩾q0.00025$, the maximum values of $p_{L1}^{*}$ in the cases of liver tissue exceed those of water.

**Table 2:** Parameters and resultant values for five different initial void fractions $\alpha_{0}$, including the cases in Fig. 7, Fig. 8. The absolute values of $\Pi_{21}$ and $\Pi_{3}$ are shown because they are originally negative. Maximum value of $p_{L1}^{*}$ is at $\tau^{*}=10\;\mu s$.

	$\alpha_{0}=0.0001$ (Fig. 7)		$\alpha_{0}=0.00025$ (Fig. 8)		$\alpha_{0}=0.0005$		$\alpha_{0}=0.00075$		$\alpha_{0}=0.001$	
	Water	Liver tissue	Water	Liver tissue	Water	Liver tissue	Water	Liver tissue	Water	Liver tissue
Nonlinear coefficient $\Pi_{1}$ [-]	3.60	3.44	3.60	3.44	3.60	3.44	3.60	3.43	3.60	3.43
Dissipation coefficient $\left|\Pi_{21}\right|$ [-]	0.0126	0.0104	0.0200	0.0165	0.0282	0.0233	0.0346	0.0285	0.0399	0.0328
Dissipation coefficient $\Pi_{22}$ [-]	0.135	0.0883	0.214	0.140	0.303	0.197	0.371	0.241	0.428	0.278
Dispersion coefficient $\left|\Pi_{3}\right|$ [-]	0.241	0.173	0.381	0.274	0.539	0.387	0.660	0.473	0.761	0.546
Phase velocity $U^{*}$ [m/s]	1197	1336	757	845	536	598	437	488	379	423
Maximum value of $p_{L1}^{*}$ [kPa]	493	360	506	560	373	505	365	438	347	384

### 4.3 Generation of higher harmonic

The dispersion effect is represented in the form of the third derivative with coefficient $\Pi_{3}$ in the resultant KZK Eq. (102) and obtained by introducing the bubble oscillation [10], [93], [25], [26], [28], [29], [27], [115], [88], [114], [95], [89], [90], [91], [92], [94], [102], [103], [113]. By the dispersion effect, the propagation speed of waves shifts depending on the frequency of each wave. Hence when the dispersion effect is indroduced, higher harmonic components generated by the nonlinear effect will propagate at a speed different from the fundamental frequency component.

**Fig. 9:**
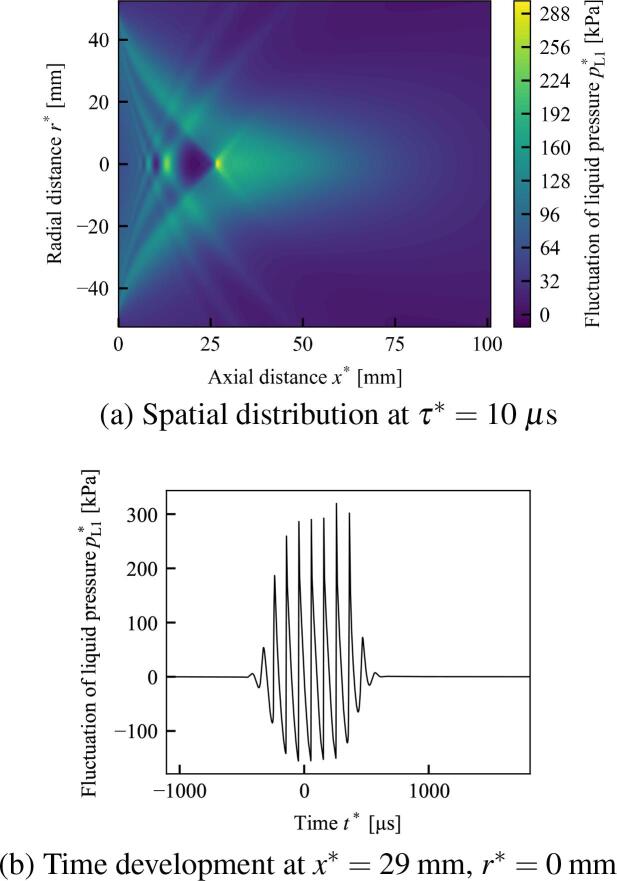
The case in which only the dispersion coefficient is set as zero and the other conditions are same as Fig. 8.

Fig. 9 shows the numerical solution that only the dispersion coefficient $\Pi_{3}$ is virtually set as zero to clarify the dispersion effect. In Fig. 7, Fig. 8b with the dispersion effect, some peak points with low values newly appeared among the discontinuous points owing to the dispersion effect. In contrast to Fig. 7, Fig. 8b, the time development of Fig. 9b does not have the new peak points. In addition, tha spatial distribution of Fig. 9a without dispersion effect has the narrower peak region of pressure rise than the Fig. 7, Fig. 8a.

### 4.4 Effect of elasticity on higher harmonic generation

In Fig. 7, the initial void fraction $\alpha_{0}=0.00025$ and the liquid is water. The frequency domains of Figs. 7f show that the fundamental frequency component gradually shift into higher harmonic components. In $x^{*}=29\;\mathrm{mm}$ and $x^{*}=32\;\mathrm{mm}$ of Fig. 7, Fig. 7, the third harmonic components ecxeed the second harmonic components.

In Fig. 8, the initial void fraction $\alpha_{0}=0.0005$ and the liquid is liver tissue. The other conditions including the frequency, the pressure amplitude and the radius of sound source, are same as Fig. 7. The maximum values of pressure fluctuations are 506 kPa and 505 kPa in Fig. 7, Fig. 8, respectively, these values are quite close. As Fig. 7, the frequency domains of Figs. 8f show that the fundamental frequency component gradually shift into higher harmonic components, however, third harmonic components do not exceed the second harmonic components in contrast to Fig. 7. In addition, Figs. 8f retain more fundamental frequency components than Figs. 7f. These results imply that the elasticity of the liquid phase supresses the generation of higher harmonic components and promotes the remnant of the fundamental frequency components; therefore, the elasticity of the liquid phase suppresses shock wave formation in practical applications.

## 5 Conclusion

Weakly nonlinear propagation of focused ultrasound in viscoelastic liquid containing multiple bubbles was investigated using the volumetric averaged equations of liquid containing multiple bubbles based on the mixture model [96], [97], [98], [99]. The viscoelasticity of the entire liquid was introduced to the momentum conservative equation in Eq. (4) and modeled with the Zener model in Eq. (16), whereas that of the bubble–liquid interface was considered in the Keller–Miksis equation in Eq. (17).

As a result of the theoretical analysis based on perturbation expansion with the multiple-scales method [121], the KZK equation in Eq. (102) describing the weakly nonlinear propagation in viscoelastic liquids containing multiple bubbles was derived. The resultant KZK Eq. (102) is composed of terms representing nonlinear, dissipation, dispersion, and diffraction effects of ultrasound propagation. As shown in Fig. 3, the liquid elasticity decreases the magnitudes of nonlinear coefficient $\Pi_{1}$, dissipation coefficients $\Pi_{21}$ and $\Pi_{22}$, and dispersion coefficient $\Pi_{3}$, and increases the phase velocity $U^{*}$ and linear natural frequency of bubble oscillation $\omega_{B}^{*}$. As shown in Fig. 4, the comparison among the viscoelastic models; Zener model, Maxwell model, and Kelvin–Voigt model, is conducted. Then, the effects of the rigidity is quite larger than the relaxation time. In addition, a comparison of the liquid viscoelasticity of the entire liquid and bubble–liquid interface was conducted. As shown in Fig. 5, the nonlinear coefficient $\Pi_{1}$ was decreased by both elasticities. However, the magnitudes of the dissipation coefficients $\Pi_{21}$ and $\Pi_{22}$, dispersion coefficient $\Pi_{3}$, phase velocity $U^{*}$, and linear natural frequency of the bubble oscillation $\omega_{B}^{*}$ were strongly affected by the elasticity of the bubble–liquid interface, while the effects of the elasticity of the entire liquid were considerably small.

In Section 4, the numerical solution of the newly obtained KZK equation was shown for different cases of the initial void fraction $\alpha_{0}$. The dispersion effect introduced by the bubble oscillation are shown, by virtually setting the dispersion coefficient $\Pi_{3}=0$ of Fig. 9. In addition, a frequency analysis was carried out using FFT and the generation of higher harmonic components was compared for water and liver tissue. As shown in the frequency analysis of Figs. 7–8f, the elasticity of liver tissue supresses the generation of higher harmonic components and promotes the remnant of the fundamental frequency components compare to the case of water, although the maximum values of $p_{L1}^{*}$ are quite close. Therefore, this result implies that the elasticity of the liquid phase suppresses shock wave formation in practical applications.

In our future work, theoretical extensions of the KZK equation will be conducted, such as incorporating blood vessels [132], [133], [134], phase change [107], [109] and heat transfer [110] across bubble–liquid interface, and bubble–bubble interaction [104], [105], [106], [46], [44], [39]. Deriving the Westervelt equation as a generalization of the present KZK equation is effective. On the other hand, incorporating the nonlinear dissipation [124], [125], [126], [127] will change the resultant equation from KZK equation and Westervelt equation, however will be very important. Ultimately, verifying the present KZK equation by comparison with experimental and direct numerical simulations is necessary.

## CRediT authorship contribution statement

**Shunsuke Kagami:** Software, Validation, Formal analysis, Investigation, Data curation, Writing - original draft, Writing - review & editing. **Tetsuya Kanagawa:** Conceptualization, Methodology, Formal analysis, Investigation, Writing - original draft, Writing - review & editing, Supervision, Project administration, Funding acquisition.

## Declaration of Competing Interest

The authors declare that they have no known competing financial interests or personal relationships that could have appeared to influence the work reported in this paper.

## Appendix A Inhomogeneous terms in Eq. (92)–(97)

The inhomogeneous terms $K_{i}\;(i=1,\ldots ,6)$ are given as

(A.1) $$K_{1}=3\frac{\partial \alpha_{1}R_{1}}{\partial t_{0}}+3\delta \left(x_{1}\right)\frac{\partial R_{1}}{\partial t_{0}}-6\frac{\partial R_{1}^{2}}{\partial t_{0}}-\frac{\partial \alpha_{1}}{\partial t_{1}}+3\frac{\partial R_{1}}{\partial t_{1}}-\frac{\partial \alpha_{1}u_{x1}}{\partial x_{0}}-\delta \left(x_{1}\right)\frac{\partial u_{x1}}{\partial x_{0}}+3\frac{\partial R_{1}u_{x1}}{\partial x_{0}}-\frac{\partial u_{x1}}{\partial x_{1}}-\left\{\begin{array}{cc} \Gamma_{r}\left(\frac{u_{r1}}{r_{1/2}}+\frac{\partial u_{r1}}{\partial r_{1/2}}\right) & (\mathrm{for}2\mathrm{Dcase}),\\ \Gamma_{y}\frac{\partial u_{y1}}{\partial y_{1/2}}+\Gamma_{z}\frac{\partial u_{z1}}{\partial z_{1/2}} & (\mathrm{for}3\mathrm{Dcase}), \end{array}\right)$$

(A.2) $$K_{2}=\left(1-\alpha_{0}\right)\frac{\partial \rho_{L1}}{\partial t_{0}}-\alpha_{0}\frac{\partial \alpha_{1}}{\partial t_{1}}-\alpha_{0}\frac{\partial \alpha_{1}u_{x1}}{\partial x_{0}}-\alpha_{0}\delta (x_{1})\frac{\partial u_{x1}}{\partial x_{0}}+\left(1-\alpha_{0}\right)\frac{\partial u_{x1}}{\partial x_{1}}+\left\{\begin{array}{cc} \Gamma_{r}\left(1-\alpha_{0}\right)\left(\frac{u_{r1}}{r_{1/2}}+\frac{\partial u_{r1}}{\partial r_{1/2}}\right) & (\mathrm{for}2\mathrm{Dcase}),\\ \left(1-\alpha_{0}\right)\left(\Gamma_{y}\frac{\partial u_{y1}}{\partial y_{1/2}}+\Gamma_{z}\frac{\partial u_{z1}}{\partial z_{1/2}}\right) & (\mathrm{for}3\mathrm{Dcase}), \end{array}\right)$$

(A.3) $$K_{3}=\alpha_{0}\frac{\partial \alpha_{1}u_{x1}}{\partial t_{0}}+\alpha_{0}\delta (x_{1})\frac{\partial u_{x1}}{\partial t_{0}}-\left(1-\alpha_{0}\right)\frac{\partial u_{x1}}{\partial t_{1}}-\frac{\partial p_{L1}}{\partial x_{1}}+\frac{\partial \tau_{\mathrm{xx}1}}{\partial x_{1}}-\left(1-\alpha_{0}\right)\frac{\partial u_{x1}^{2}}{\partial x_{0}}-\frac{2}{3}\mu_{L}\frac{\partial^{2}u_{x1}}{\partial x_{0}^{2}}+2\left(K_{e,L}-\frac{2}{3}G_{L}\right)\frac{\partial^{2}d_{x1}}{\partial x_{0}\partial x_{1}}+\left\{\begin{array}{c} \Gamma_{r}\left(K_{e,L}-\frac{2}{3}G_{L}\right)\left(\frac{1}{r_{1/2}}\frac{\partial d_{r1}}{\partial x_{0}}+\frac{\partial d_{r1}}{\partial r_{1/2}\partial x_{0}}\right)\\ +\Gamma_{r}\left(\frac{\tau_{\mathrm{xr}1}}{r_{1/2}}+\frac{\partial \tau_{\mathrm{xr}1}}{\partial r_{1/2}}\right)(\mathrm{for}2\mathrm{Dcase}),\\ \left(K_{e,L}-\frac{2}{3}G_{L}\right)\left(\Gamma_{y}\frac{\partial d_{y1}}{\partial y_{1/2}\partial x_{0}}+\Gamma_{z}\frac{\partial d_{z1}}{\partial z_{1/2}\partial x_{0}}\right)\\ +\Gamma_{y}\frac{\partial \tau_{\mathrm{xy}1}}{\partial y_{1/2}}+\Gamma_{z}\frac{\partial \tau_{\mathrm{xz}1}}{\partial z_{1/2}}(\mathrm{for}3\mathrm{Dcase}), \end{array}\right)$$

(A.4) $$K_{4}=-3\left(\kappa -1\right)\frac{\partial R_{1}}{\partial t_{1}}-\frac{\partial T_{G1}}{\partial t_{1}}-3\left(\kappa -1\right)\left(3\kappa -4\right)R_{1}\frac{\partial R_{1}}{\partial t_{0}}-3\left(\kappa -1\right)\frac{\partial T_{G1}R_{1}}{\partial t_{0}}-3\left(\kappa -1\right)u_{x1}\frac{\partial R_{1}}{\partial x_{0}}-u_{x1}\frac{\partial T_{G1}}{\partial x_{0}}-\lambda_{G}T_{G1},$$

(A.5) $$K_{5}=\Delta^{2}\frac{\partial^{2}R_{1}}{\partial t_{0}^{2}}-V\Delta \frac{\partial p_{L1}}{\partial t_{0}}+\left[-4G_{L}+3\left(\kappa -2\right)p_{G0}-\frac{\Delta^{2}}{\Omega^{2}}\right]R_{1}^{2}+3p_{G0}R_{1}T_{G1}-4\lambda_{\mathrm{relax},L}G_{L}\frac{\partial R_{1}}{\partial t_{0}}+4\mu_{L}\frac{\partial R_{1}}{\partial t_{0}},$$

(A.6) $$K_{6}=-\lambda_{\mathrm{relax},L}\frac{\partial \tau_{\mathrm{xx}1}}{\partial t_{0}}+2\mu_{L}\frac{\partial u_{x1}}{\partial x_{0}}+2G_{L}\frac{\partial d_{x1}}{\partial x_{1}}$$
